# Influence of Excess Weight on the Risk of SARS-CoV-2 Infection and Hospitalization: A Case–Control Study in a Rural Area of Spain

**DOI:** 10.3390/healthcare14142080

**Published:** 2026-07-12

**Authors:** Mónica Álvarez Ruiz, Francisco Javier Pérez-Rivas

**Affiliations:** 1 Gerencia de Asistencia Sanitaria de Soria, 42005 Soria, Spain; malvarezruiz@saludcastillayleon.es; 2 Departamento de Enfermería, Facultad de Enfermería, Fisioterapia y Podología, Universidad Complutense de Madrid, 28040 Madrid, Spain; 3 Grupo de Investigación UCM “Salud Pública–Estilos de Vida, Metodología Enfermera y Cuidados en el Entorno Comunitario”, Departamento de Enfermería, Facultad de Enfermería, Fisioterapia y Podología, Universidad Complutense de Madrid, 28040 Madrid, Spain; 4 Instituto de Investigación Sanitaria Hospital 12 de Octubre (Imas12), 28041 Madrid, Spain

**Keywords:** COVID-19, obesity, body mass index, infection risk, hospitalization, rural population

## Abstract

**Background/Objectives**: This study aimed to examine whether excess body weight is associated with susceptibility to SARS-CoV-2 infection, and to explore its relationship with COVID-19 severity, measured as the risk of hospitalization, in a rural population in Spain. **Methods**: A case–control observational study was conducted in the Soria Health Area, including 236 confirmed COVID-19 cases and 243 controls without a previous diagnosis of COVID-19. In addition, a nested cohort analysis was performed among the cases to evaluate the association between excess body weight and disease severity, defined by the need for hospitalization. Bivariate and multivariable logistic regression analyses were performed to identify factors independently associated with SARS-CoV-2 infection and hospitalization. **Results**: Excess body weight was not independently associated with SARS-CoV-2 infection risk after multivariable adjustment. In contrast, increasing body mass index was strongly associated with COVID-19 severity. Among individuals with confirmed infection, hospitalization risk increased nearly fivefold in individuals with overweight (OR = 4.97; 95% CI: 1.75–16.22), and eightfold in those with grade I obesity (OR = 8.35; 95% CI: 2.56–29.83). The highest odds ratio was observed for grade II–III obesity (OR = 72.00; 95% CI: 6.31–820.84), although this estimate was based on a small number of events and should therefore be interpreted with caution. These associations remained significant after multivariable adjustment. **Conclusions**: In this rural setting, excess body weight was not linked to an increased risk of SARS-CoV-2 infection but remained independently associated with greater COVID-19 severity after adjustment for potential confounding factors. The graded association between BMI and hospitalization risk highlights obesity as a key modifier of adverse outcomes and supports preventive interventions into clinical practice for patients with excess body weight.

## 1. Introduction

In December 2019, health authorities in Wuhan reported more than twenty cases of pneumonia of unknown etiology with documented epidemiological links, including seven severe cases [1]. On 7 January 2020, the causative agent was identified as a novel coronavirus belonging to the Coronaviridae family, subsequently designated SARS-CoV-2 [1]. On 30 January 2020, the World Health Organization (WHO) recognized this outbreak as a Public Health Emergency of International Concern [2], and on 11 March 2020, it was formally declared a global pandemic [3].

As of November 2025, over 779 million cases of COVID-19 have been reported globally, including approximately 282 million in Europe and nearly 14 million in Spain [4].

SARS-CoV-2 infection is asymptomatic or mild in most cases; however, approximately 10% of infected individuals develop severe disease, frequently progressing to acute respiratory distress syndrome associated with a massive release of proinflammatory mediators [5]. Several demographic and clinical factors have been identified as determinants of disease severity [6]. Epidemiological studies consistently report that individuals with pre-existing respiratory disease, cancer, or other chronic conditions present a higher risk of complications, admission to Intensive Care Units (ICUs), and COVID-19-related mortality [7,8].

Among the chronic conditions most extensively studied, obesity represents a highly prevalent disease that has been associated with an increased risk of complications related to COVID-19 infection [9,10]. The prevalence of obesity has tripled worldwide over the past four decades, and if current trends persist, it is estimated that by 2030, 38% of the global population will be overweight and 20% will be obese [11]. Chronic overnutrition and obesity are associated with metabolic alterations characterized by increased leptin secretion, local inflammation, and the sustained release of inflammatory mediators. This chronic inflammatory state represents a well-established risk factor for the development of diseases such as diabetes mellitus, cancer, and cardiovascular disorders [11].

Previous evidence supports the consideration of obesity as a relevant risk factor in SARS-CoV-2 infection, as shown in studies conducted in New York and France [8,12]. In a cohort of 5279 patients within a New York health system, obesity was identified, along with advanced age, as the most important factor associated with hospitalization and critical illness, defined as ICU admission, need for mechanical ventilation, or death [8]. Similarly, Simonnet et al. conducted a study in France including all consecutive patients admitted to the ICU of the Roger Salengro Hospital for COVID-19 pneumonia, in which obesity was significantly associated with disease severity, with a particularly pronounced effect among patients with a body mass index (BMI) ≥ 35 kg/m^2^ [12].

Moreover, several studies have shown that patients with COVID-19 and higher BMI values tend to have a worse clinical prognosis on average [13]. Obesity-related conditions, such as diabetes mellitus, have also been associated with an increased risk of complications. In this regard, a study by Orioli et al. reported a significant association between diabetes and the development of metabolic complications, including diabetic ketoacidosis, leading to an increased risk among patients with COVID-19 and diabetes [14]. In addition, a higher proportion of visceral adipose tissue has been linked to a greater frequency of ICU admissions. Taken together, these findings reinforce the hypothesis that obesity, and particularly visceral adiposity, contributes to a more unfavorable clinical course of SARS-CoV-2 infection.

During the pandemic, and up to November 2025, more than 7.1 million deaths due to COVID-19 have been reported worldwide, approximately 121,000 of which occurred in Spain [15]. Notably, most studies on obesity and COVID-19 prevalence at both national and international levels have focused on areas with high population density, whereas sparsely populated regions have been comparatively underrepresented. Research conducted in rural areas is particularly relevant due to differences in access to healthcare systems, services, and infrastructure compared with urban settings. Studies focused on rural populations may help raise awareness of the adverse effects of obesity among both the general population and healthcare professionals, promote active patient identification, support the implementation of appropriate preventive and therapeutic interventions, and encourage health policymakers to allocate additional resources to health education initiatives.

Therefore, the aim of this study was to analyze the influence of excess body weight on SARS-CoV-2 infection and COVID-19 severity in a rural population in Spain.

## 2. Materials and Methods

An analytical observational study with a case–control design was conducted to evaluate the association between excess body weight and SARS-CoV-2 infection. Among diagnosed cases, a nested cohort analysis was performed to assess the relationship between excess body weight and COVID-19 severity, defined as the need for hospital care.

The study was carried out in the Soria Health Area (Spain), which has a population of 91,048 inhabitants. The study population was divided into a case group and a control group. Cases included individuals with a confirmed positive test for active SARS-CoV-2 infection between 1 January and 31 January of 2022, corresponding to the sixth pandemic wave. The control group comprised individuals without a previous diagnosis of COVID-19, selected to have sociodemographic characteristics similar to those of the cases in terms of sex, age, and Basic Health Zone.

Inclusion criteria were residence in the Soria Health Area, random selection, confirmed SARS-CoV-2 infection (case) or absence of prior infection (control), and voluntary participation following the provision of informed consent.

Exclusion criteria included inability to establish telephone contact and inability to understand the study information or questionnaire due to language barriers or cognitive impairment.

Sample size calculation [16] was based on an estimated SARS-CoV-2 infection prevalence of 18.6% according to the scientific literature [17], with a 95% confidence level and a 5% margin of error. Assuming a potential loss rate of 30%, an adjusted sample size of 295 participants was estimated. Consequently, an initial random sample of 300 cases was selected from the database of patients with a positive SARS-CoV-2 test in January 2022 (N = 7805). Given the lack of sufficient local data on obesity prevalence at the time of study design, sample size estimation was pragmatically based on infection prevalence. This approach allowed the definition of an adequate number of participants to ensure representativeness of the study population and to detect clinically relevant differences between cases and control groups.

For the control group, 389 individuals were selected through stratified random sampling, representing approximately a 30% increase compared with the number of cases to compensate for potential losses and possible reclassification during follow-up. This sample was drawn from a database of 56,340 individuals who had not been diagnosed with COVID-19 as of 31 January 2023. Telephone interviews with control participants were conducted between February and July 2023.

The dependent variable for the assessment of infection risk was having COVID-19 (yes/no), whereas disease severity was evaluated based on the need for hospital care. In this study, we considered age, sex, height, weight, Body Mass Index (BMI), and the need for hospital care as the main variables. Age was recorded in years as a continuous variable, and sex was classified as female or male. Height (in meters) and weight (in kilograms) were used to calculate BMI. The need for hospital care was initially documented in five categories: no hospital care; visit to the emergency department or hospital stay of fewer than 3 days; admission lasting 3 to 7 days; admission longer than 7 days; and admission to the ICU. For the purposes of the analysis, this variable was subsequently recoded into a binary outcome (yes/no) to facilitate the analysis of disease severity.

The study population was identified using the database of the Urban COVID Unit of the Soria Health Area (Spain). A list of all patients who tested positive for COVID-19 between 1 January and 31 January 2022 (N = 7805) was obtained, from which a random sample was selected to establish the case group. A pilot survey was conducted in March 2022, and telephone interviews with cases were carried out between April and June 2022.

Subsequently, using Medora, the electronic primary care medical record system in Castilla y León (northwestern Spain), the Basic Health Zone of each case participant was identified to ensure proportional distribution when selecting the control group. From the same database, a list of individuals who had not been diagnosed with COVID-19 up to 31 January 2023 (N = 56,340) was obtained. Controls were selected one year after the case inclusion period to ensure that they had remained free of a documented SARS-CoV-2 infection during the entire observation period. This approach minimized the risk of including individuals who were in the incubation period or who developed COVID-19 shortly after the recruitment of the case group, thereby reducing outcome misclassification. A stratified random sampling by Basic Health Zone was then performed to constitute the control group.

The Medora electronic primary care medical record system was used to identify eligible participants, determine their corresponding Basic Health Zone, and verify that control participants had not been diagnosed with SARS-CoV-2 infection before study inclusion. Anthropometric data (height and weight) and the remaining epidemiological variables were collected through structured telephone interviews using a questionnaire specifically designed for this study. A detailed description of all variables, their definitions, classifications, and response categories is provided in Supplementary Table S1.

Some variables were grouped prior to analysis to facilitate statistical interpretation. BMI was categorized according to WHO criteria [18,19,20] as normal weight (18.5–24.9 kg/m^2^), overweight (25.0–29.9 kg/m^2^), obesity grade I (30.0–34.9 kg/m^2^), obesity grade II (35.0–39.9 kg/m^2^), and obesity grade III (≥40 kg/m^2^). For participants younger than 18 years, BMI classification was performed according to the World Health Organization (WHO) age- and sex-specific BMI reference standards appropriate for each age group. The WHO Child Growth Standards were used for children younger than 5 years, whereas the WHO Growth Reference for children and adolescents aged 5–19 years was applied to participants aged 5 years and older. The hospital care variable was recoded into a binary outcome (yes/no) to facilitate statistical analysis. This decision was based on the limited number of participants within the individual hospital care categories, particularly ICU admissions and prolonged hospitalizations, which would have resulted in sparse data and unstable estimates in the inferential analyses.

A descriptive analysis of the sample was performed. For quantitative variables (age, height, weight, BMI), the Kolmogorov–Smirnov test was used to assess normality. For variables without a normal distribution, the median, range, first and third quartiles, and interquartile range were calculated, whereas for normally distributed variables, the mean, standard deviation, and range were reported. Qualitative variables were described using absolute frequencies and percentages, stratified by case and control groups.

To analyze factors associated with SARS-CoV-2 infection, a bivariate analysis was conducted between the dependent variable (case/control) and the independent variables. The chi-square test was used for qualitative variables, Student’s t test for normally distributed quantitative variables, and the Mann–Whitney U test for non-normally distributed quantitative variables. Following peer-review, the comparison of BMI between participants with and without hospital care was additionally verified using the Mann–Whitney U test to confirm the robustness of the results. Odds ratios (ORs) with 95% Confidence Intervals (95% CI) were calculated to estimate the magnitude of associations.

Similarly, a second bivariate analysis was performed to evaluate factors associated with disease severity, using hospital care (yes/no) as the dependent variable and applying the same statistical tests. A significance level of *p* < 0.05 was established. Participants classified as underweight (n = 6) were excluded from inferential analysis due to their small number and the absence of hospitalized cases. Additionally, due to the limited number of hospitalized cases in obesity grades II and III, these categories were combined for inferential analysis.

In addition, multivariable logistic regression analyses were performed to identify the health determinants independently associated with both SARS-CoV-2 infection and hospitalization. Candidate variables included the demographic and biological characteristics, comorbidity-related factors, lifestyle factors, social and community networks, and socioeconomic and cultural conditions evaluated in the study. Variables considered clinically relevant or associated with the outcome in the bivariate analysis were entered into the forward likelihood ratio procedure. Adjusted odds ratios (aORs) and 95% confidence intervals (95% CIs) were calculated. Model performance was evaluated using a confusion matrix, comparing the observed and predicted classifications of the dependent variable. The overall classification accuracy was calculated as the proportion of correctly classified cases.

Data were recorded using Microsoft Excel (Microsoft Corporation, Redmond, WA, USA) and analyzed using IBM SPSS Statistics for Windows, version 25.0 (IBM Corp., Armonk, NY, USA).

The study was approved by the Burgos and Soria Research Ethics Committee (2671). All procedures involving human participants were conducted in accordance with the Declaration of Helsinki. Informed consent was obtained from all participants.

No generative artificial intelligence tools were used in the design of the study, data collection, data analysis, interpretation of results, or preparation of the scientific content of this manuscript.

This study is reported in accordance with the Strengthening the Reporting of Observational Studies in Epidemiology (STROBE) guidelines.

## 3. Results

**Figure 1:**
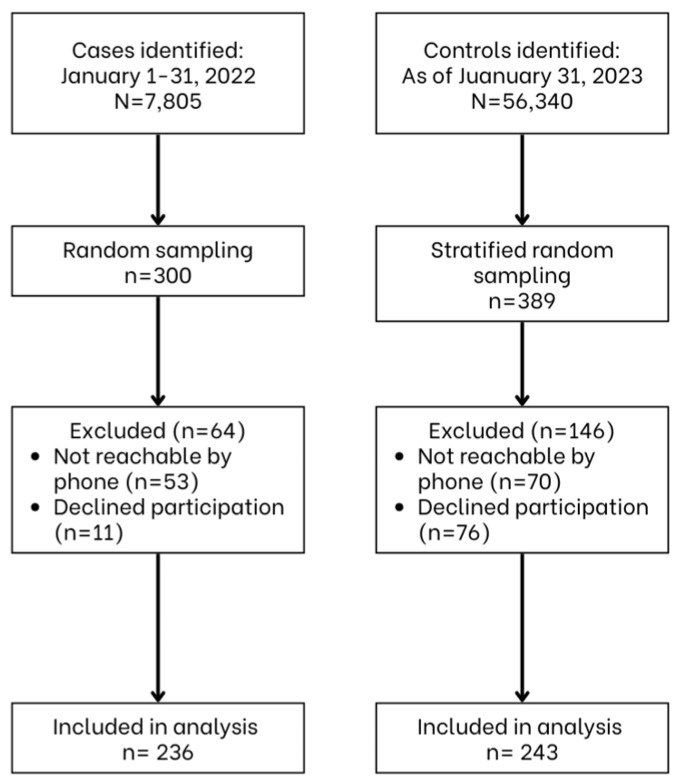
Flowchart of the participant selection and participation process.

From the reference population of the province of Soria, 689 subjects were initially selected (300 cases and 389 controls). The participation rate was 78.6% in the case group and 62.4% in the control group. The final sample comprised 479 participants, including 236 confirmed cases of SARS-CoV-2 infection and 243 controls without a prior diagnosis of COVID-19. Figure 1 presents the flow diagram of the selection and participation process from the reference population to the final analytical sample.

The mean age of the sample was 38.7 ± 24.5 years, and 55.1% of participants were women. The main sociodemographic and anthropometric characteristics of cases and controls are shown in Table 1.

No relevant differences were observed in the distribution of age or height between groups. The proportion of women was slightly higher among cases. Body weight showed an identical median in both groups (65 kg), with similar interquartile ranges. Mean BMI was comparable between cases and controls, remaining around 23 kg/m^2^ with a similar standard deviation.

When BMI distribution was examined according to the WHO classification, normal weight predominated in both groups (approximately 53%), followed by overweight (around 30%). When compared with data from the general Spanish population provided by the Spanish National Statistics Institute (INE) [21], the study sample showed a higher proportion of normal-weight individuals and a lower prevalence of overweight compared with the Spanish population, while obesity prevalence was similar. BMI distribution was comparable between cases and controls, with minor differences across weight categories.

Regarding the dependent variable reflecting the need for hospitalization, Table 2 presents the absolute and relative frequency distribution.

Overall, 11.9% of participants with confirmed SARS-CoV-2 infection required hospitalization (Table 2). The results of the inferential analyses evaluating factors associated with obesity in relation to infection risk and disease severity are presented below.

### 3.1. Influence of Excess Body Weight on SARS-CoV-2 Infection

In the first phase of inferential analysis, the association between excess body weight and the probability of SARS-CoV-2 infection was evaluated. A bivariate analysis (Table 3) was performed between BMI categories and case status (COVID case: yes/no). No statistically significant association was observed between BMI and the risk of SARS-CoV-2 infection (α = 0.05). To confirm these findings, BMI was also analyzed as a continuous variable using an independent-samples t-test (Table 3). Similarly, no significant differences were found in mean BMI between cases and controls (*p* = 0.385).

A multivariable logistic regression analysis was subsequently performed to evaluate the independent association between health determinants and the risk of SARS-CoV-2 infection. The final model achieved an overall classification accuracy of 82.7%. After adjustment for the health determinants included in the model, BMI was not independently associated with the risk of SARS-CoV-2 infection, confirming the findings of the bivariate analysis.

### 3.2. Influence of Excess Body Weight on COVID-19 Severity

In the second phase of the analysis, we examined the relationship between excess body weight and the severity of SARS-CoV-2 infection, using the need for hospital care as an indicator. Among participants with confirmed COVID-19 (n = 236), 11.9% required hospitalization. A progressive increase in hospitalization risk was observed as BMI increased. Compared with individuals with normal weight, overweight was associated with an almost fivefold higher odds of requiring hospital admission (OR 4.97; 95% CI 1.75–16.22; *p* = 0.004), and grade I obesity with an eightfold higher odds (OR 8.35; 95% CI 2.56–29.83; *p* = 0.001). Among participants with obesity grades II–III, hospitalization risk was higher (OR = 72.00; 95% CI: 6.31–820.84; *p* = 0.001), although the limited sample size restricts the precision of this estimate (Table 4). These results support a biological gradient between BMI and COVID-19 severity, with hospitalization risk increasing progressively across obesity categories.

When BMI was analyzed as a quantitative variable, similar findings were observed. Participants who required hospital care had a substantially higher mean BMI than those who were not hospitalized (29.42 vs. 23.19; *p* < 0.001), which is consistent with the pattern seen across BMI categories and reinforces the presence of a relationship between excess body weight and COVID-19 severity (Table 4). To confirm the robustness of this finding, a Mann–Whitney U test was additionally performed, yielding consistent results (U = 1306.0, Z = −4.735, *p* < 0.001).

A multivariable logistic regression analysis was subsequently performed to identify the health determinants independently associated with the need for hospital care among patients with confirmed SARS-CoV-2 infection. The final model achieved an overall classification accuracy of 94.1%. After adjustment for the health determinants included in the model, overweight and grade II obesity remained independently associated with the need for hospital care, whereas grade I obesity showed a borderline association. In addition, fatigue, dyspnoea, pneumonia, and mask use with non-household members were also identified as independent predictors of hospital care. The adjusted odds ratios and 95% confidence intervals for the variables retained in the final model are presented in Table 5.

## 4. Discussion

In this rural population sample, no association was observed between excess body weight and the risk of SARS-CoV-2 infection; however, a biological gradient relationship was identified between BMI and COVID-19 severity, assessed as the need for hospital care. Moreover, a gradient was observed whereby higher BMI values were associated with higher odds of developing complications. The multivariable logistic regression analysis confirmed that excess body weight remained independently associated with the need for hospital care after adjustment for the health determinants included in the model.

The findings of the present study are consistent with previous evidence linking obesity to a higher probability of complications following SARS-CoV-2 infection. Although these associations have been widely reported, our study contributes additional epidemiological evidence by externally validating these associations in a rural primary care population, a setting that remains underrepresented in the literature. Furthermore, unlike many previous studies that focused exclusively on disease severity, we simultaneously evaluated the relationship between excess body weight and both the risk of SARS-CoV-2 infection and the need for hospital care, providing a broader assessment of the role of excess body weight across different stages of COVID-19. In a study conducted in Navarra (Spain), Fresán et al. reported that severe obesity doubled the risk of hospitalization (aRR 2.20) and severe disease (aRR 2.30), defining severe disease as ICU admission or death; this association was particularly pronounced among individuals younger than 50 years (aRR 13.80) [22]. Similarly, the meta-analysis by Poly et al., including 543,399 patients, showed an increased risk of mortality among individuals with obesity (RR 1.42) and a progressive rise in the risk of death according to obesity class (grade I: RR 1.27; grade II: RR 1.56; grade III: RR 1.92) [23]. In addition, early meta-analyses reported by Yang already indicated a higher risk of severe COVID-19 among individuals with obesity, with patients presenting significantly higher BMI and an approximately twofold increased risk of severe disease (pooled OR = 2.31) [24]. This association was subsequently confirmed and strengthened in an updated meta-analysis including a larger number of studies and participants, which reported increased odds of hospitalization (OR = 1.54) and ICU admission (OR = 1.48), including the need for mechanical ventilation and death (OR = 1.48) [25]. Overall, the available evidence supports BMI as a marker of vulnerability to adverse outcomes in SARS-CoV-2 infection and is consistent with a biological gradient between increasing BMI and clinical severity across diverse populations.

Evidence from studies focused on hospitalized patients provides similar conclusions. In Canada, Plourde et al. observed that a 10 kg/m^2^ increase in BMI was associated with a 3.5-fold higher risk of in-hospital mortality after accounting for age and comorbidities [26]. In a study among U.S. veterans, Eastment reported that class III obesity increased the risk of mechanical ventilation and mortality, particularly in individuals younger than 65 years [27]. Both studies highlight obesity as an independent risk factor, not solely mediated by associated comorbidities.

Furthermore, fat distribution appears to play a relevant role in disease progression. Using computed tomography, Földi et al. demonstrated that visceral adiposity was significantly higher among patients that required intensive care or invasive ventilation [28]. Thus, available evidence reinforces the role of excess adipose tissue—particularly central adiposity—as a clinically relevant determinant of COVID-19 severity.

Several biological mechanisms may explain the greater susceptibility and severity observed in individuals with obesity. Ectopic adipose tissue can amplify the inflammatory response through increased expression of cytokines such as interleukin-6 (IL-6) and Tumor Necrosis Factor alpha (TNF-α), together with elevated angiotensin II levels and prothrombotic mediators [29,30]. In parallel, lower concentrations of adiponectin—an anti-inflammatory adipokine—have been described, and its reduction has been associated with increased angiotensin II levels [31]. Obesity has also been linked to overexpression of ACE2 receptors, which may facilitate viral entry and act as a viral reservoir; together with oxidative stress, these mechanisms may contribute to lung injury, endothelial dysfunction, and the hypercoagulable state characteristic of severe COVID-19 [32,33]. Collectively, these pathophysiological processes support the higher frequency of hospitalization and complications observed in individuals with obesity and underscore the importance of integrating metabolic assessment into preventive and clinical management strategies.

One of the main strengths of this study is that it provides evidence from a rural population, a setting that remains underrepresented in the obesity and COVID-19 literature. This approach broadens existing knowledge, which has largely been derived from urban areas and large hospital centers, and offers a more contextualized view of the impact of obesity in low-density areas. In addition, the multivariable analysis allowed adjustment for a broad range of health determinants, strengthening the evidence that excess body weight is independently associated with the need for hospital care. The relatively high participation rate and the use of verified clinical records strengthen the internal validity and reliability of the findings.

Nevertheless, several limitations should be considered. Given the observational design, a causal relationship between obesity and COVID-19 severity cannot be established. It is possible that some individuals in the control group experienced asymptomatic infection, which may have attenuated the observed associations. The sample size was calculated based on the estimated prevalence of SARS-CoV-2 infection rather than obesity prevalence because reliable local data on obesity were not available at the time of study design. Although this approach ensured an adequate sample for the primary objective of the study, the study was not specifically powered to evaluate COVID-19 severity. Consequently, the relatively small number of participants requiring hospital care may have limited both the statistical precision and the stability of the multivariable estimates, particularly in the highest BMI categories. Therefore, these findings should be interpreted with appropriate caution and confirmed in larger studies specifically powered to evaluate COVID-19 severity. Another limitation is that height and weight were self-reported during the telephone interview and were not validated against electronic medical records. Although the Medora system was used to identify participants and confirm the absence of previous SARS-CoV-2 infection among controls, anthropometric data could not be verified because participants had not provided specific consent to access their complete electronic medical records. Self-reported measurements may be affected by recall or social desirability bias, potentially leading to an underestimation of BMI and misclassification into lower WHO BMI categories. Any resulting misclassification would likely be non-differential and therefore tend to attenuate rather than overestimate the observed associations. A further limitation is that COVID-19 severity was assessed using hospital care as a binary outcome. Although hospital care is a widely used indicator of disease severity in epidemiological studies, dichotomizing this variable may have reduced the ability to distinguish between different levels of clinical severity, such as short hospital stays, prolonged hospitalization, or intensive care unit admission. Moreover, hospital admission may be influenced by healthcare organization, local clinical protocols, and healthcare resource availability. Therefore, hospital care should be interpreted as a pragmatic surrogate marker of disease severity rather than a direct measure of clinical severity. Another limitation is the temporal difference between the recruitment of cases and controls. Cases were identified during the sixth pandemic wave (January 2022), whereas controls were selected in 2023 after confirming the absence of a documented SARS-CoV-2 infection throughout the observation period and were subsequently interviewed by telephone. This strategy was adopted to minimize the risk of misclassification of controls. However, changes in circulating SARS-CoV-2 variants, vaccination coverage, and population immunity over time may have partially reduced the epidemiological comparability between cases and controls and should be considered when interpreting the findings. If present, this type of misclassification would most likely have biased the association towards the null by reducing the contrast between cases and controls. Finally, the small size of certain subgroups limits the statistical power to detect differences in the most extreme BMI categories. Accordingly, the estimate observed for grade II–III obesity should be interpreted with caution because it was based on a small number of hospitalized participants and showed limited precision. Despite these limitations, the study provides results consistent with the available evidence and contributes to understanding the role of obesity in the clinical course of COVID-19, particularly in rural contexts.

## 5. Conclusions

In this study, no association was observed between excess body weight and an increased risk of SARS-CoV-2 infection; however, excess body weight remained independently associated with greater clinical severity of COVID-19 after adjustment for the health determinants included in the model. Higher body mass index values were significantly associated with the need for hospitalization, showing a risk gradient proportional to increasing BMI. These findings support previous evidence identifying obesity as a key determinant in the clinical course of COVID-19 and reinforce the importance of integrating anthropometric assessment into prevention and clinical management strategies. From a clinical practice perspective, prioritizing follow-up and intervention in patients with excess body weight through health promotion and obesity prevention programs may contribute to reducing the impact of future respiratory infections. Further studies are needed to confirm these findings in different population settings and to evaluate the effectiveness of preventive interventions targeted at this high-risk group.

## Figures and Tables

**Table 1:** Sociodemographic and anthropometric characteristics by study group (cases and controls).

Variable	Total (n = 479)	Cases (n = 236)	Controls (n = 243)
Sex, n (%)			
Female	264 (55.1)	139 (58.9)	125 (51.4)
Male	215 (44.9)	97 (41.1)	118 (48.6)
Age, years	37 [18–56]	37 [17–53]	37 [20–59]
Height, m	1.63 [1.53–1.72]	1.63 [1.53–1.72]	1.63 [1.53–1.74]
Weight, kg	65 [52–75]	65 [52.5–75]	65 [51–76]
BMI, mean (SD)	23.72 ± 5.41	23.94 ± 5.49	23.51 ± 5.33
BMI (WHO classification), n (%)			
Underweight	16 (3.3)	6 (2.5)	10 (4.1)
Normal weight	254 (53.0)	125 (53.0)	129 (53.1)
Overweight	143 (29.9)	70 (29.7)	73 (30.0)
Obesity grade I	53 (11.1)	31 (13.1)	22 (9.1)
Obesity grade II	9 (1.9)	3 (1.3)	6 (2.5)
Obesity grade III	4 (0.8)	1 (0.4)	3 (1.2)

Quantitative variables are presented as median [Q1–Q3], except BMI, which is reported as mean ± SD. The sample includes participants younger than 18 years.

**Table 2:** Distribution of hospital care among confirmed SARS-CoV-2 cases.

		CASES (236)
		N (%)
Hospital Care	No	208 (88.14)
	Yes, <3 days (ED visit/admission)	21 (8.90)
	Yes, 3 days to 1 week	3 (1.27)
	Yes, >1 week	4 (1.69)
	Yes, ICU admission	0 (0)
Hospital Care (recoded)	Yes (any hospital care)	28 (11.86)
	No	208 (88.14)

**Table 3:** Association between BMI (qualitative and continuous variables) and SARS-CoV-2 infection by study group (cases and controls).

**BMI**	**Cases n (%)**	**Controls n (%)**	**OR (95% CI)**	***p*** **Value**
**WHO classification**				
Underweight	6 (2.54)	10 (4.12)	0.62 (0.20–1.72)	0.367
Normal weight ^1^	125 (52.97)	129 (53.09)		
Overweight	70 (29.66)	73 (30.04)	0.99 (0.66–1.49)	0.960
Obesity grade I	31 (13.14)	22 (9.05)	1.45 (0.80–2.67)	0.221
Obesity grade II	3 (1.27)	6 (2.47)	0.52 (0.11–2.00)	0.357
Obesity grade III	1 (0.42)	3 (1.23)	0.34 (0.02–2.73)	0.358
**BMI (continuous)**	**Cases (n = 236)**	**Controls (n = 243)**	**t**	***p*** **Value**
Mean ± SD (kg/m^2^)	23.94 ± 5.49	23.51 ± 5.33	0.870	0.385
Underweight	15.14 ± 2.52	15.42 ± 2.86		
Normal weight ^1^	20.88 ± 2.99	20.95 ± 2.99		
Overweight	26.50 ± 2.92	25.83 ± 3.34		
Obesity grade I	30.25 ± 4.42	30.09 ± 4.30		
Obesity grade II	32.67 ± 8.05	29.90 ± 8.16		
Obesity grade III	56.88 ± 0	42.17 ± 0.68		

^1^ Reference value: normal weight.

**Table 4:** Association between BMI and COVID-19–related hospitalization among confirmed cases.

**BMI**	**Hospital Care** **Yes n (%)**	**Hospital Care** **No n (%)**	**OR (95% CI)**	***p*** **Value**
**WHO classification**				
Normal weight ^1^	5 (17.86)	120 (57.70)	Reference	–
Overweight	12 (42.86)	58 (27.89)	4.97 (1.75–16.22)	0.004
Obesity grade I	8 (28.58)	23 (11.06)	8.35 (2.56–29.83)	0.001
Obesity grade II–III	3 (10.70)	1 (0.49)	72.00 (6.31–820.84)	0.001
**BMI (Continuous)**	**Hospital Care Yes (n = 28)**	**Hospital Care No (n = 208)**	**t**	***p*** **Value**
Mean ± SD (kg/m^2^)	29.43 ± 7.31	23.20 ± 4.76	6.049	<0.001
Normal weight ^1^	21.91 ± 1.37	20.84 ± 3.03		
Overweight	26.76 ± 2.70	26.44 ± 2.98		
Obesity grade I	32.73 ± 1.65	29.38 ± 4.77		
Obesity grade II–III	43.82 ± 11.35	23.43 ± 0		

^1^ Reference value: normal weight. Participants classified as underweight (n = 6) were excluded from the inferential analysis because no hospitalized participants were observed in this category.

**Table 5:** Multivariable logistic regression analysis of factors independently associated with hospital care among patients with confirmed SARS-CoV-2 infection.

Variable	B	Sig.	Exp(B)	95% C.I. for Exp(B)	
				Lower	Upper
Mask use with non-household members	1.869	0.004	6.484	1.815	23.163
Fatigue	1.420	0.028	4.135	1.169	14.624
Dyspnoea	1.857	0.002	6.403	2.019	20.303
Pneumonia	3.736	0.007	41.929	2.722	645.978
Recoded BMI		0.013			
Recoded BMI: Overweight	1.596	0.021	4.935	1.275	19.105
Recoded BMI: Obesity grade I	1.506	0.055	4.508	0.967	21.005
Recoded BMI: Obesity grade II	5.832	<0.001	341.104	13.444	8654.488
Constant	−5.787	<0.001	0.003		

## Data Availability

The raw data supporting the conclusions of this article will be made available by the authors on request.

## Supplementary Materials

The following supporting information can be downloaded at: https://www.mdpi.com/article/10.3390/healthcare14142080/s1, Table S1. Definition and Classification of the Variables Included in the Study.

## Abbreviations

The following abbreviations are used in this manuscript:

aRR	Adjusted Relative Risk
BMI	Body Mass Index
CI	Confidence Intervals
ICUs	Intensive Care Units
INE	National Statistics Institute
Kg	Kilograms
m	meter
ORs	Odds ratios
RECM	Research Ethics Committee for Medicines
RR	Relative Risk
SPSS	Statistical Package for the Social Sciences
STROBE	Strengthening the Reporting of Observational Studies in Epidemiology
TNF-α	Tumor Necrosis Factor alpha
WHO	World Health Organization
