# Supplementary material for: Influence of Excess Weight on the Risk of SARS-CoV-2 Infection and Hospitalization: A Case–Control Study in a Rural Area of Spain
Source: Healthcare (Basel). 2026 Jul 12;14(14):2080. doi: 10.3390/healthcare14142080 (PMC13411857; doi:10.3390/healthcare14142080)
Supplement: Supplementary file 1 [file healthcare-14-02080-s001.zip › healthcare-4392365-supplementary.pdf]

Supplementary Table S1. Definition and Classification of the Variables Included in the Study

A. Demographic and Biological Variables

Variable	Type	Categories
Sex	Categorical (binary)	Female; Male
Age (years)	Continuous	—
Race	Categorical (nominal)	Mongoloid; American; Caucasian; Malayan; Ethiopian
Blood group	Categorical (nominal)	A+, A−, B+, B−, AB+, AB−, O+, O−; Unknown/No response
Height (m)	Continuous	—

B. Comorbidity-Related Variables

Variable	Type	Categories
Weight (kg)	Continuous	—
Body mass index (BMI)	Continuous	—
Disability	Categorical (ordinal)	No recognized disability; ≤33%; 34–64%; ≥65%
Chronic disease	Categorical (binary)	Yes; No
Chronic medication	Categorical (binary)	Yes; No
Number of chronic medications	Continuous	—
Diabetes mellitus	Categorical (binary)	Yes; No
Hypertension	Categorical (binary)	Yes; No
Hypercholesterolemia	Categorical (binary)	Yes; No
Chronic obstructive pulmonary disease (COPD)	Categorical (binary)	Yes; No
Asthma	Categorical (binary)	Yes; No
Cardiovascular disease	Categorical (binary)	Yes; No
Kidney disease	Categorical (binary)	Yes; No
Liver disease	Categorical (binary)	Yes; No
Cancer	Categorical (binary)	Yes; No
Type of cancer	Categorical (nominal)	Recorded according to the reported diagnosis
Number of chronic diseases	Discrete	None; 1; 2; 3; 4; 5; 6; More than 6

C. Individual Lifestyle Variables

Variable	Type	Categories
Smoking status	Categorical (nominal)	Current smoker; Never smoker; Former smoker
Number of cigarettes per day	Continuous	—
Smoking duration	Continuous	—
Alcohol consumption frequency	Categorical (ordinal)	Never; Once/month or less; 2–4/month; 2–3/week; ≥4/week
Number of alcoholic drinks per occasion	Categorical (ordinal)	0; 1–2; 3–4; 5–6; 7–9; ≥10
Frequency of consuming >6 standard drinks	Categorical (ordinal)	Never; Less than monthly; Monthly; Weekly; Daily/almost

		daily
Aerobic exercise	Categorical (binary)	Yes; No
Balance exercise	Categorical (binary)	Yes; No
Muscle-strengthening exercise	Categorical (binary)	Yes; No
Frequency of attending bars	Categorical (ordinal)	<2/month; 2–3/week; 4–7/week
Mask use outdoors	Categorical (binary)	Yes; No
Mask use at work	Categorical (binary)	Yes; No
Mask use with non-household members	Categorical (binary)	Yes; No
Mask use on public transport	Categorical (binary)	Yes; No
Exposure to SARS-CoV-2	Categorical (binary)	Yes; No
Close contact with a confirmed case	Categorical (binary)	Yes; No
COVID-19 vaccination status	Categorical (nominal)	One dose; Two or more doses; Unvaccinated
Willingness to receive COVID-19 vaccination	Categorical (binary)	Yes; No

D. Social and Community Network Variables

Variable	Type	Categories
Marital status	Categorical (nominal)	Married/Partner; Single; Separated; Widowed
Family support	Categorical (ordinal)	Strongly disagree; Disagree; Neither agree nor disagree; Agree; Strongly agree
Social support	Categorical (ordinal)	Strongly disagree; Disagree; Neither agree nor disagree; Agree; Strongly agree

E. Socioeconomic and Cultural Variables

Variable	Type	Categories
Nationality	Categorical (nominal)	Recorded according to participant's nationality
Educational level	Categorical (ordinal)	Illiterate; Primary; Secondary; High school/Vocational training (Level I); Vocational training (Level II); University
Occupation	Categorical (nominal)	Unemployed; Teleworking; Non-customer-facing occupation; Retail worker; Law enforcement; Education; Healthcare/social care professional; Retired; Other
Net monthly income	Categorical (ordinal)	<€600; €601–900; €901–1200; €1201–1800; €1801–2400; >€2400; No response
Waiting time for medical care	Categorical (ordinal)	<24 h; 24–72 h; 72 h–1 week; >1 week; Unknown/No response/Not required

F. SARS-CoV-2 Infection-Related Variables

Variable	Type	Categories
Reason for SARS-CoV-2 diagnostic testing	Categorical (nominal)	Population screening; Symptoms; Close contact with a confirmed case
Suspected setting of infection	Categorical (nominal)	Unknown; Household; Social; Workplace; School; Healthcare/social care facility
Symptoms	Categorical (nominal)	Fever; Chills; Cough; Odynophagia; Severe fatigue; Muscle pain; Dyspnoea; Headache; Nausea/vomiting; Diarrhoea; Anosmia; Ageusia; Skin rash; Asymptomatic
Timing of symptom onset	Categorical (nominal)	No symptoms; Before diagnosis; After diagnosis
Pneumonia	Categorical (binary)	Yes; No
Face-to-face medical assessment	Categorical (binary)	Yes; No
Hospital care	Categorical (ordinal)	No hospital care; <3 days; 3 days–1 week; >1 week; ICU admission
COVID-19 vaccination	Categorical (binary)	Yes; No
Isolation	Categorical (binary)	Yes; No
Number of close contacts	Continuous	—
Number of SARS-CoV-2-positive close contacts	Continuous	—
